# Influence of overnight orthokeratology lens fitting decentration on corneal topography reshaping

**DOI:** 10.1186/s40662-018-0100-7

**Published:** 2018-03-15

**Authors:** Jiaojie Chen, Wei Huang, Rong Zhu, Jun Jiang, Yiyu Li

**Affiliations:** 1 School of Optometry and Ophthalmology, WenZhou Medical University, WenZhou, ZheJiang China; 2 Eye Hospital of Wenzhou Medical University, 270 West Xueyuan Road, Wenzhou, Zhejiang 325027 China

**Keywords:** Orthokeratology, Lens fitting decentration, Zernike coefficients, Contact lens, Corneal topography

## Abstract

**Background:**

This retrospective study was designed to investigate the sole influence of orthokeratology (OK) lens fitting decentration on the Zernike coefficients of the reshaped anterior corneal surface.

**Methods:**

This study comprised a review of 106 right eyes and measurements of corneal topography both before OK and at 1-month follow-up visit. A routine was designed to calculate local corneal surface astigmatism and assist the determination of OK lens fitting decentration from pupil center. The pupil-centered corneal Zernike coefficients of baseline (PCCB) and post-treatment (PCCP) were calculated. Meanwhile, the OK-lens-centered corneal Zernike coefficients of post-treatment (OCCP) were also calculated and considered as the presumptive ideal fitting group without decentration. Relationships between lens fitting decentration and the change of Zernike coefficients including (PCCP − PCCB) and (PCCP − OCCP) were analyzed.

**Results:**

Patients with a mean age of 11 ± 2.36 years old had an average spherical equivalent refractive error of −3.52 ± 1.06 D before OK. One month after treatment, OK lens fitting decentration from pupil center was 0.68 ± 0.35 mm. RMS of 3rd-order (*P* < 0.05), RMS of 4th-order (*P* < 0.001) and RMS of total high order (*P* < 0.001) corneal Zernike coefficients were increased in PCCP by comparing with OCCP, which was solely caused by lens fitting decentration. Nevertheless, no significant difference was observed in ${C}_2^0$ (*P* > 0.05). For the high order corneal Zernike coefficients in (PCCP – OCCP), radial distance of decentration was correlated with ${C}_3^{-1}$ (*r* = −0.296, *P* < 0.05), ${C}_3^1$ (*r* = −0.396, *P* < 0.001), and ${C}_4^0$ (*r* = 0.449, *P* < 0.001), horizontal decentration was significantly correlated with ${C}_3^1$ (*r* = 0.901, *P* < 0.001) and ${C}_5^1$ (*r* = 0.340, *P* < 0.001), and vertical decentration was significantly correlated with ${C}_3^{-1}$ (*r* = 0.904, *P* < 0.001).

**Conclusions:**

OK lens fitting decentration within 1.5 mm hardly influenced the change of corneal spherical power for myopia correction, but significantly induced additional corneal high order Zernike coefficients including ${C}_3^{-1}$, ${C}_3^1$, ${C}_4^0$, and ${C}_5^1$.

## Background

Modern orthokeratology (OK) is a clinical nonsurgical method for temporary myopia correction and even controlling myopic progress in adolescents [1–6]. With professional inspection, clinical monitoring and careful personal hygiene management, the safety of overnight OK treatment has repeatedly been confirmed [6–9]. Overnight OK lens reshapes cornea surface by flattening the central cornea and relatively steepening the mid-peripheral cornea for the desired myopia correction [1, 10–12]. Corneal reshaping may also lead to a decreased thickness of the central cornea with an increased thickness of the mid-peripheral cornea [1, 11–14]. It has been proved that myopia correction with OK lens is mainly attributed to anterior corneal surface reshaping [12, 15–17]. However, a slight but statistically significant change on the posterior corneal surface in the early phase of OK progress was also reported [15, 16].

Corneal reshaping results in the disparity of corneal surface power between the central flattened corneal region and the annular surrounding region. It is easy, however not precise enough, to identify the central treatment zone (TZ) based on corneal curvature maps or power maps provided by computer-assisted video-keratography [18–22]. The estimated displacement from pupil center to the nominal TZ center represents lens fitting decentration.

The change of corneal topography as well as corneal and ocular aberrations induced by OK can be influenced by lens fitting decentration [18–22]. Previous studies focused on the difference of corneal surface or wavefront [20, 23–29] between baseline and post-treatment, therefore demonstrating the joint effect of corneal reshaping and lens fitting decentration that commonly happened simultaneously. Since the ideal fitting case without any decentration is impracticable in the clinic, it is still challenging to answer the following question: How to clarify the sole influence of OK lens fitting decentration on corneal topography reshaping within the pupil aperture? The best answer to this question relies on the construction of intended corneal surface reshaped under an ideal fitting condition.

The modal Zernike approach has been extensively used for the representation of corneal topography [30–32] and ocular wavefront [33, 34] because of its simple analytical form for surface curvature and power extraction as well as visual quality evaluation. In this paper, we described corneal surface morphology with Zernike coefficients and proposed a novel corneal surface astigmatism map to identify the TZ of OK on the cornea and the lens fitting decentration. Two subregions were taken from the reshaped corneal surface, which were the pupil-centered corneal region and the TZ-centered corneal region. The TZ-centered corneal region was considered to mostly mimic the presumptive ideal corneal surface reshaped under a perfect fitting condition. Comparison of corneal Zernike coefficients between the two areas was performed for the first time to reveal the individual effect of OK lens fitting decentration on corneal topography reshaping.

## Methods

### Subjects

A total of 106 subjects (36 males and 70 females), ranged from 7 to 21 years old, mean 11 ± 2.36 years, were reviewed in this retrospective study. The subjects were all treated at the Eye Hospital of Wenzhou Medical University and were asked to wear OK lenses no fewer than 8 h per night. The inclusion criteria comprised a spherical refractive error of less than −6.00 DS with refractive astigmatism of −1.50 DC or less, best-corrected distance visual acuity of logMAR (logarithm of the minimum angle of resolution) 0.0 or better before treatment, and radial distance of OK lens fitting decentration of less than 1.5 mm to prevent sclera from being covered. Additionally, no OK lens wear in the last six months, no contact lens wear within at least one-month pre-treatment, no current ocular or systemic disease, and no use of medications that might influence refractive error. All subjects were treated according to the tenets of the Declaration of Helsinki.

### Orthokeratology lens and lens fitting

We selected two popular brands of the OK lens with the universal and similar four-curve design concept. One of them was the Lucid OrthoK lens, and the other was the Euclid OrthoK lens for overnight wear. Lucid OrthoK lenses (Lucid Korea Co Ltd., South Korea) were manufactured with the hexafocon-A material (Boston XO, DK = 100 × 10^− 11^[cm^2^/s][ml O_2_/ml × mm Hg], Polymer Technology Corporation). Euclid OrthoK lenses (Euclid Systems Corporation, America) were manufactured with Boston Equalens II material (DK = 90 × 10^− 11^ [cm^2^/s][ml O_2_/ml × mm Hg], Polymer Technology Corporation). All these lenses possess a reverse-geometry design. The overall diameter range of OK lenses is 10.2 mm to 11.2 mm, and the central thickness is 0.22 mm to 0.23 mm.

All of these patients were treated by the doctor who had been working in the field of OK treatment at the Eye Hospital for over ten years. The most suitable trial lens was selected based on the corneal topography and the desired visual acuity for the initial lens-wearing trial. According to the fitting evaluation under corneal fluorescein pattern, a micro-adjustment would be made during the following trials. The final lens was ordered by the doctor through manufacturer’s guidelines combined with parameters of the most suitable trial lens.

### Corneal topography

All the patients took a detailed list of ocular examinations including slit-lamp evaluation, fluorescein staining, subjective refraction and corneal topography both before OK lens fitting and at 1-month follow-up visit. Corneal profiles were measured with Medmont E300 Video-Keratography (Medmont International Pty Ltd., Victoria, Australia, Model E300 U) by a specialized technician within one hour after OK lens removal. And each of the profiles was the best-focus image (the accuracy greater than 95%) from the four frames which were captured automatically. The Medmont E300 uses Placido rings to map corneal surface and provides the extrapolated topography data over a maximum ring diameter of 12 mm with 2500 discrete output points uniformly spaced with the subgrid size of 0.24 mm × 0.24 mm.

### Lens fitting decentration and corneal Zernike coefficients

Unlike the conventional methods that utilized corneal axial curvature, tangential curvature, or mean curvature to assist the determination of lens fitting decentration [18–22, 35], the new method proposed here has used corneal surface astigmatism map calculated from the principal curvatures of the anterior corneal surface with the theory of differential geometry [36–38].

First, the measured anterior corneal surface within an aperture of 10 mm in diameter was fitted using Zernike polynomials expansions [30–34] defined in Cartesian coordinate with the radial order of *n* and angular frequency of *m*. A large number of Zernike terms (up to an order of 16) were required to improve fitting accuracy due to the freeform features of the cornea. The achieved RMS fit error covering all the data points was less than 0.03 μm.

At each point of corneal surface *S* = *S*(*x, y*), principal curvatures, denoted *κ*_1_ and *κ*_2_, which are maximum and minimum curvatures in the normal planes were solved with analytic method [36]:

1 $$ \left( EG-{F}^2\right){\upkappa}^2-\left( EN+ GL-2 FM\right)\upkappa +\left( LN-{M}^2\right)=0 $$

$$ E=1+{S}_x^2,\kern0.5em F={S}_x{S}_y,\kern0.5em G=1+{S}_y^2,\kern0.5em L=\frac{S_{xx}}{\sqrt{1+{S}_x^2+{S}_y^2}},\kern0.5em M=\frac{S_{xy}}{\sqrt{1+{S}_x^2+{S}_y^2}},\kern0.5em N=\frac{S_{yy}}{\sqrt{1+{S}_x^2+{S}_y^2}} $$

where *S*_*x*_, *S*_*y*_, *S*_*xx*_ and *S*_*xy*_ are the first and second derivatives along the horizontal and vertical directions, and *S*_*xy*_ is the crossed second derivative.

The directions of normal plane where the curvature takes its maximum and minimum values are always perpendicular, if *κ*_1_ does not equal *κ*_2_, and are called the principal directions determined by

2 $$ {\alpha}_i=\arctan \left(-\frac{M-{\kappa}_iF}{N-{\kappa}_iG}\right),\kern0.5em i=1,2 $$

Principal powers were then defined as the product of principal curvatures and the difference of refractive indexes of the medium separated by the corneal surface. The difference of two principal powers at each point represented the local corneal surface astigmatism (LCSA) with axis given by principal direction *α*_1_ of the high power *κ*_1_.

**Fig. 1:**
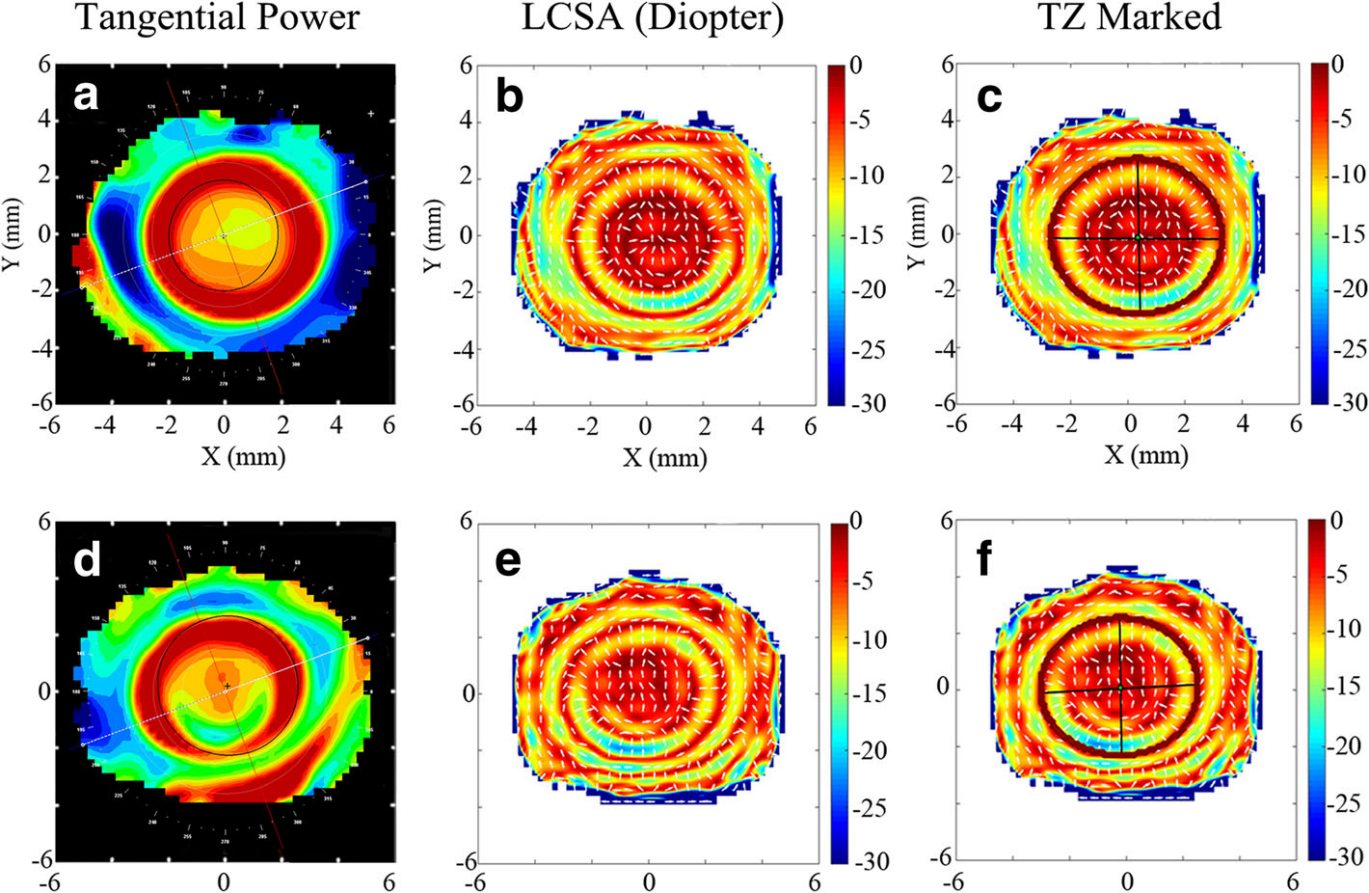
Comparison between tangential power and LCSA for two subjects. **a** Tangential power, **b** LCSA and **c** LCSA with TZ marked of subject-I; **d** Tangential power, **e** LCSA and **f** LCSA with TZ marked of subject-II

Figure 1 shows both the tangential power map supplied by the Medmont E300 and the proposed LCSA map which demonstrated the considerable corneal irregular astigmatism [39] across the reshaped cornea surface. During the overnight OK treatment, OK lens wear forced central cornea region to be flattened and mid-peripheral cornea region to be relatively steepened, thereby generating the minimum LCSA in the transition zone connecting the two areas. The dark elliptic rings depicted in Fig. 1c and Fig. 1f highlight the trajectory of minimum LCSA (being close to zero diopter), meanwhile represent the effective boundary between base curve and reverse curve of OK lens fitting on the cornea. The ring was elliptical, not circular in shape because of the inherent corneal spherocylinder.

Corneal region encircled by this elliptic ring represented the central TZ of OK on the cornea and was called the OK-lens-centered cornea region. Lens fitting decentration was the displacement from the center of the elliptic ring to the pupil center. Temporal displacement and inferior displacement from pupil center mean a negative horizontal lens fitting decentration and a negative vertical lens fitting decentration, respectively.

**Fig. 2:**
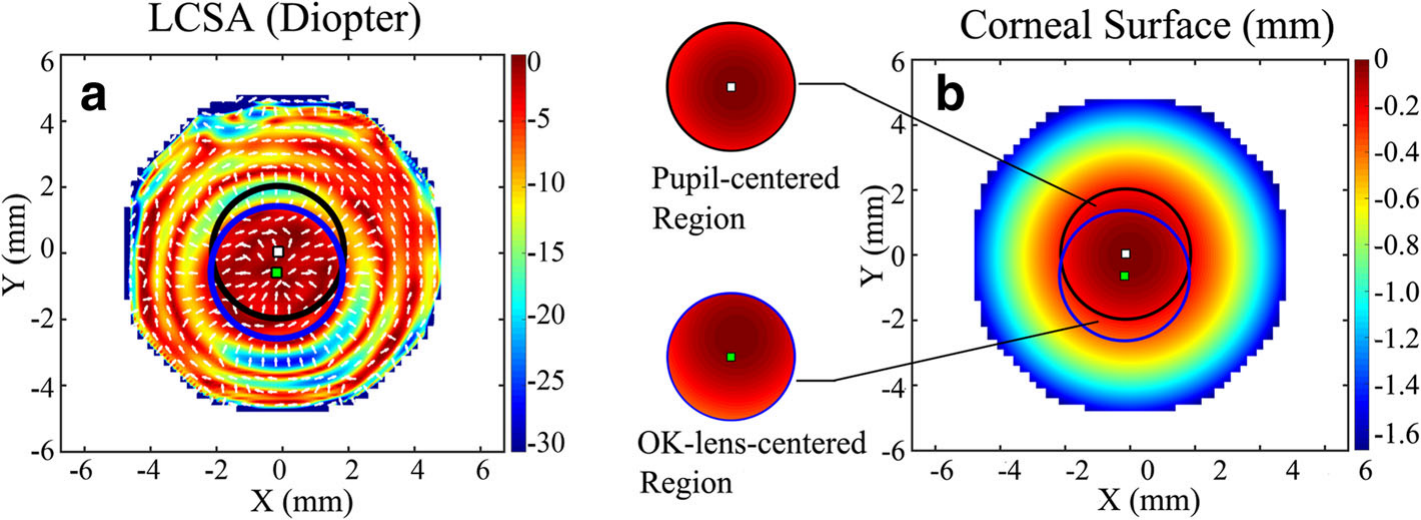
Pupil-centered corneal region and OK-lens-centered corneal region of an OK-lens-treated subject-III. **a** Two subregions were marked on LCSA map, black circle: pupil-centered region; blue circle: OK-lens-centered region. **b** The two subregions were separated from the reshaped corneal surface

Two subregions with an aperture size of 4 mm in diameter were then taken from the reshaped corneal surface, which comprised the pupil-centered corneal region and the OK-lens-centered corneal region. Figure 2 shows that the two subregions were marked on LCSA map and separated from the corneal surface. Since the OK-lens-centered corneal region was always covered by the base curve of the OK lens, the corneal geometry features except piston and tilt components of this region should be insensitive to lens fitting decentration and therefore might best mimic the presumptive ideal corneal surface reshaped without fitting decentration.

In this study, all the subjects were chosen to have a lens fitting decentration of less than 1.5 mm, so that sclera would not interact with the OK lens. The two subregions separated from the reshaped corneal surface were fitted using Zernike polynomials to obtain the pupil-centered corneal Zernike coefficients of post-treatment (PCCP) and the OK-lens-centered corneal Zernike coefficients of post-treatment (OCCP), respectively. Meanwhile, the pupil-centered corneal Zernike coefficients of pre-treatment (PCCB) were also calculated as a baseline. Note that, each Zernike polynomial represented a corneal surface deformation mode.

### Statistical analysis

SPSS Statistics 18.0 (IBM Statistics, Armonk, NY) was used for statistical analysis of the lens fitting decentration and the corneal Zernike coefficients. Data from right eyes were used for analysis. Each Zernike coefficient and root-mean-square (RMS) of 3rd order, 4th order and total high order (3rd to 7th order) Zernike coefficients were compared for each pair of PCCB, OCCP and PCCP using the paired *t*-test. The difference between PCCP and PCCB and the difference between PCCP and OCCP were analyzed concerning lens fitting decentration (e.g., horizontal and vertical decentration, the radial distance of decentration) using Pearson correlation (*r*) test. A *P* value less than 0.05 was considered statistically significant.

## Results

One hundred six subjects (36 males and 70 females) were evaluated with their mean age of 11 ± 2.36 (mean ± standard deviation, range 7 to 21 years). Before treatment, spherical equivalent refractive error was −3.52 ± 1.06 D (range −1.25 to −5.75 D) with spherical refractive error of −3.35 ± 1.01 DS (range −1.00 to −5.25 DS) and astigmatism of −0.47 ± 0.38 DC (range 0.00 to −1.50 DC). After 1-month OK lens wear, 104 eyes got the uncorrected visual acuity of logMAR 0.1 or better (98.1%). Radial distance of lens fitting decentration was 0.68 ± 0.35 mm (range 0.05 to 1.49 mm), horizontal decentration was −0.40 ± 0.46 mm (range −1.24 to + 0.98 mm) and vertical decentration was −0.15 ± 0.45 mm (range −1.40 to + 0.93 mm). For horizontal displacement, there were 16 eyes (15.1%) observed a nasal decentration, and 90 eyes (84.9%) observed a temporal decentration. For vertical displacement, there were 44 eyes (41.5%) observed a superior decentration, and 62 eyes (58.5%) observed an inferior decentration. There were 52 eyes (49.1%) observed a decentration in the inferotemporal quadrant, 38 eyes (35.8%) in the superotemporal quadrant, 10 eyes (9.4%) in inferonasal quadrant, and 6 eyes (5.7%) in the superonasal quadrant.

Zernike modes near the center of Zernike pyramid, which can profoundly affect the visual performance [40–42], were chosen from PCCB, OCCP, and PCCP and listed in Table 1 including their comparisons.

**Table 1:** Analysis of corneal Zernike coefficients in PCCB, OCCP and PCCP with aperture size of 4 mm in diameter

${C}_n^m$	PCCB	OCCP	PCCP	OCCP vs. PCCB	PCCP vs. PCCB	PCCP vs. OCCP
	(mean ± standard deviation) (μm)			(Paired *t* test)		
${C}_2^0$	75.57 ± 2.40	70.96 ± 2.68	71.13 ± 2.83	0.000^*^	0.000^*^	0.095
${C}_3^{-1}$	−0.09 ± 0.21	0.01 ± 0.65	−0.28 ± 0.81	0.106	0.021^†^	0.011^†^
${C}_3^1$	0.11 ± 0.18	0.41 ± 0.65	−0.42 ± 0.87	0.000^*^	0.000^*^	0.000^*^
${C}_4^{-2}$	−0.00 ± 0.03	−0.02 ± 0.12	− 0.04 ± 0.28	0.209	0.180	0.452
${C}_4^0$	0.30 ± 0.06	0.34 ± 0.26	0.67 ± 0.40	0.126	0.000^*^	0.000^*^
${C}_4^2$	−0.03 ± 0.05	−0.00 ± 0.18	0.04 ± 0.29	0.109	0.007^†^	0.118
${C}_5^{-1}$	0.00 ± 0.03	−0.04 ± 0.12	−0.01 ± 0.22	0.000^*^	0.504	0.176
${C}_5^1$	0.01 ± 0.04	−0.01 ± 0.11	−0.07 ± 0.24	0.051	0.001^*^	0.028^†^
3^rd^ -order RMS	0.32 ± 0.17	0.93 ± 0.49	1.07 ± 0.83	0.000^*^	0.000^*^	0.038^†^
4^th^ -order RMS	0.32 ± 0.06	0.45 ± 0.22	0.80 ± 0.43	0.000^*^	0.000^*^	0.000^*^
High orders RMS	0.48 ± 0.14	1.12 ± 0.49	1.47 ± 0.90	0.000^*^	0.000^*^	0.000^*^

*Abbreviations*: *PCCB* = Pupil-centered corneal Zernike coefficients of baseline/pre-treatment; *OCCP* = OK-lens-centered corneal Zernike coefficients of post-treatment; *PCCP* = Pupil-centered corneal Zernike coefficients of post-treatment

^*^P < 0.001 (paired *t* test), significant statistical significance

^†^*P* < 0.05(paired *t* test), statistical significance

Comparison of OCCP with PCCB in Table 1 reveals that the presumptive ideal OK lens fitting (corneal reshaping without lens fitting decentration) could cause a significant decrease in ${C}_2^0$ (*P* < 0.001) and a substantial increase in RMS of 3rd-order (*P* < 0.001), RMS of 4th-order (*P* < 0.001) and RMS of high order (*P* < 0.001) Zernike coefficients, but no change in ${C}_4^0$ (*P* > 0.05).

Comparison of PCCP with PCCB in Table 1 reveals that the actual OK lens fitting (corneal reshaping combined with lens fitting decentration) resulted in a significant decrease in ${C}_2^0$ (*P* < 0.001) and a considerable increase in ${C}_4^0$ (*P* < 0.001), RMS of 3rd-order (*P* < 0.001), RMS of 4th-order (*P* < 0.001) and RMS of high order (*P* < 0.001) Zernike coefficients of pupil-centered corneal region.

Comparing PCCP with OCCP in Table 1 shows the sole effect of lens fitting decentration, which did not cause significant change in ${C}_2^0$ (*P* = 0.095 > 0.05) but a substantial increase in ${C}_4^0$ (*P* < 0.001), RMS of 3rd-order (*P* = 0.038 < 0.05), RMS of 4th-order (*P* < 0.001) and RMS of high order (*P* < 0.001) Zernike coefficients.

According to the significant difference of ${C}_3^{-1}$ (*P* = 0.021 < 0.05), ${C}_3^1$ (*P* < 0.001), ${C}_4^0$ (*P* < 0.001), ${C}_4^2$ (*P* = 0.007 < 0.05), ${C}_5^1$ (*P* < 0.001) between PCCP and PCCB (see Table 1), Table 2 lists the correlations between lens fitting decentration and the modification of Zernike coefficients calculated by (PCCP − PCCB).

**Table 2:** The correlations between lens fitting decentration and the change of Zernike coefficients in (PCCP – PCCB) and (PCCP − OCCP)

${C}_n^m$	PCCP − PCCB			PCCP − OCCP		
	Radial	Horizontal	Vertical	Radial	Horizontal	Vertical
${C}_3^{-1}$	/	/	0.693^*^	−0.296^†^	/	0.904^*^
${C}_3^1$	−0.379^*^	0.730^*^	/	−0.396^*^	0.901^*^	/
${C}_4^0$	0.531^*^	/	/	0.449^*^	/	/
${C}_4^2$	/	−0.301^†^	0.275^†^	/	/	/
${C}_5^1$	/	0.369^*^	/	/	0.340^*^	/

*Abbreviations*: *PCCP* = Pupil-centered corneal Zernike coefficients of post-treatment; *PCCB* = Pupil-centered corneal Zernike coefficients of baseline/pre-treatment; *OCCP* = OK-lens-centered corneal Zernike coefficients of post-treatment

^*^P < 0.001 (Pearson correlation (*r*) test), significant statistical significance

^†^*P* < 0.05 (Pearson correlation (*r*) test), statistical significance

According to the significant difference of ${C}_3^{-1}$ (*P* = 0.011 < 0.05), ${C}_3^1$ (*P* < 0.001), ${C}_4^0$ (*P* < 0.001), ${C}_5^1$ (*P* = 0.028 < 0.05) between PCCP and OCCP (see Table 1), similar analysis was performed to obtain the correlations between lens fitting decentration and the change of Zernike coefficients calculated by (PCCP − OCCP) in Table 2.

**Fig. 3:**
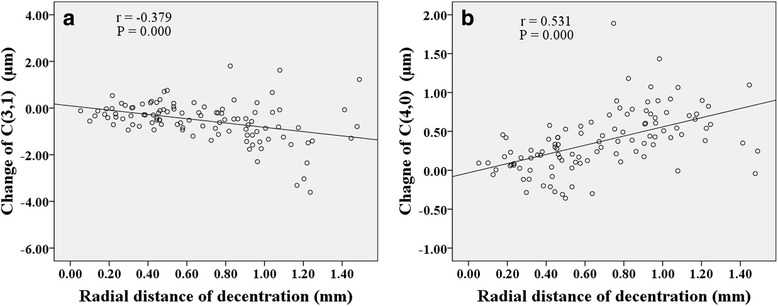
The correlations between radial distance of decentration and Zernike coefficients in (PCCP − PCCB). **a** ${C}_3^1$. **b** ${C}_4^0$

**Fig. 4:**
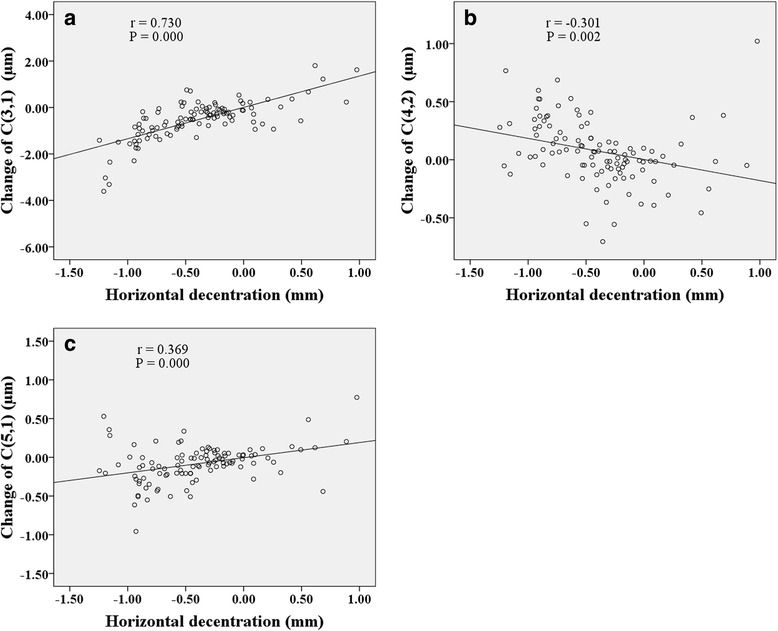
The correlations between horizontal decentration and Zernike coefficients in (PCCP−PCCB). **a** ${C}_3^1$. **b** ${C}_4^2$. **c** ${C}_5^1$

**Fig. 5:**
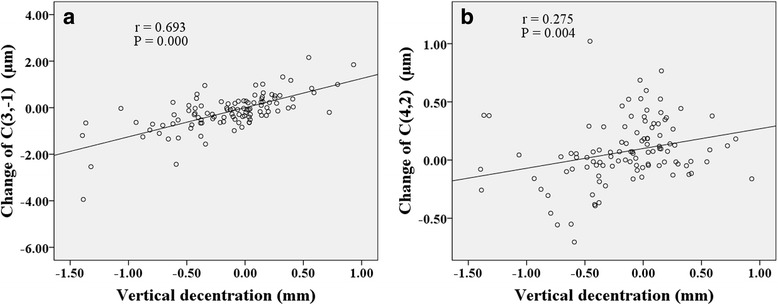
The correlations between vertical decentration and Zernike coefficients in (PCCP−PCCB). **a** ${C}_3^{-1}$. **b** ${C}_4^2$

Figures 3, 4, 5 show correlations between lens fitting decentration and the modification of Zernike coefficients calculated by (PCCP−PCCB). Radial distance of decentration was significantly correlated with ${C}_3^1$ (*r* = −0.379, *P* < 0.001) and ${C}_4^0$ (*r* = 0.531, *P* < 0.001) as shown in Fig. 3. Horizontal decentration was substantially correlated with ${C}_3^1$ (*r* = 0.730, *P* < 0.001), ${C}_4^2$ (*r* = −0.301, *P* < 0.05) and ${C}_5^1$ (*r* = 0.369, *P* < 0.001) as shown in Fig. 4. Vertical decentration was significantly correlated with ${C}_3^{-1}$ (*r* = 0.693, *P* < 0.001) and ${C}_4^2$ (*r* = 0.275, *P* < 0.05) as shown in Fig. 5.

**Fig. 6:**
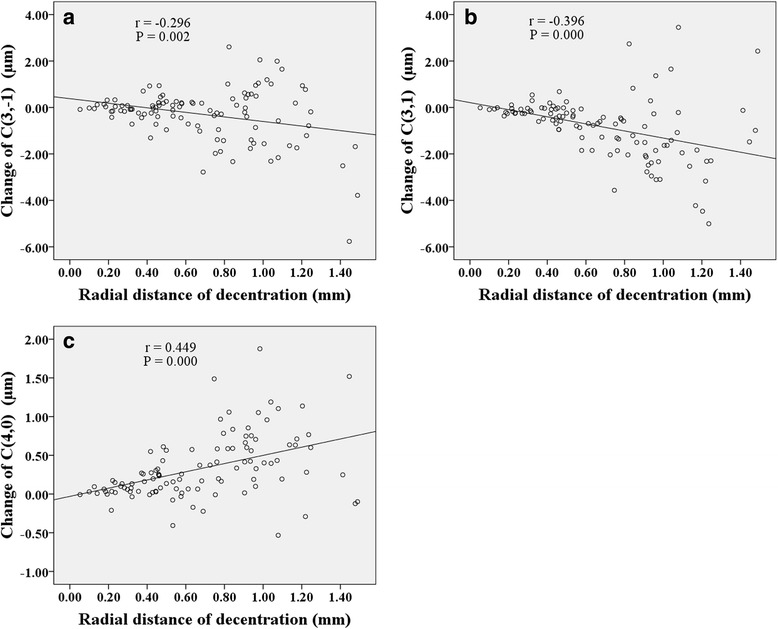
The correlations between radial distance of decentration and Zernike coefficients in (PCCP − OCCP). **a** ${C}_3^{-1}$. **b** ${C}_3^1$. **c** ${C}_4^0$

**Fig. 7:**
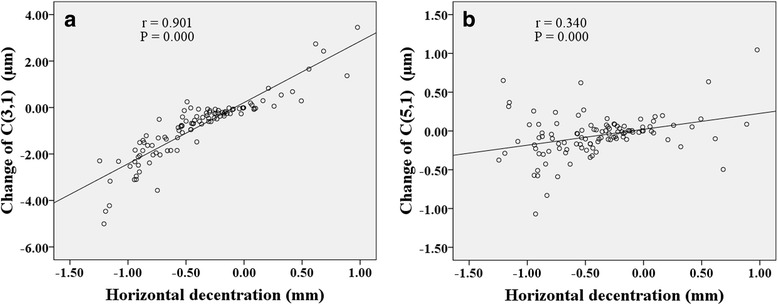
The correlations between horizontal decentration and Zernike coefficients in (PCCP − OCCP). **a** ${C}_3^1$. **b** ${C}_5^1$

**Fig. 8:**
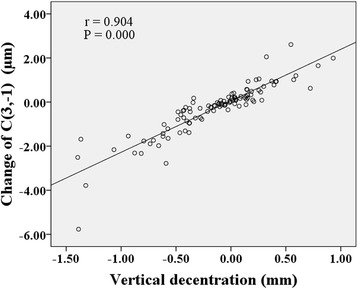
The correlation between vertical decentration and Zernike coefficient ${C}_3^{-1}$ in (PCCP − OCCP)

Figures 6, 7, 8 show correlations between lens fitting decentration and the change of Zernike coefficients determined by (PCCP−OCCP). Radial distance of decentration was significantly correlated with ${C}_3^{-1}$ (*r* = −0.296, *P* < 0.05), ${C}_3^1$ (*r* = −0.396, *P* < 0.001), and ${C}_4^0$ (*r* = 0.449, *P* < 0.001) as illustrated in Fig. 6. Horizontal decentration was significantly correlated with ${C}_3^1$ (*r* = 0.901, *P* < 0.001) and ${C}_5^1$ (*r* = 0.340, *P* < 0.001) as shown in Fig. 7. Vertical decentration was appreciably correlated with ${C}_3^{-1}$ (*r* = 0.904, *P* < 0.001) as shown in Fig. 8.

## Discussion

Owing to the effectiveness of controlling myopic progress in adolescents [1–6], there is an increasing prevalence of OK treatment that has been chosen by more than 1.5 million adolescents in China [6]. Fortunately, the safety of OK lens treatment also has repeatedly been confirmed both for short-term and long-term therapy [6–9]. However, fitting decentration cannot be avoided entirely during the procedure. In our study, we investigated the sole influence of overnight OK lens fitting decentration on corneal topography with our new method, differing from the previous study dealing with the joint effect of corneal reshaping and lens fitting decentration [20, 23–29].

To accurately calculate the OK lens fitting decentration on the cornea, LCSA maps were proposed for the first time to our best knowledge to assist the determination of OK-lens-centered corneal region or the TZ of OK. Contrasting the previous methods using surface power or surface curvature [18–22, 35], a distinct trajectory of minimum corneal surface astigmatism could be observed on LCSA map, which facilitated the accurate identification of OK lens position and even the working boundaries of the segmented curves of the OK lens on the cornea.

By comparing the Zernike coefficients calculated for the pupil-centered corneal surface and the OK-lens-centered corneal surface, the divergence between PCCP and PCCB demonstrated the combined effect of corneal reshaping and lens fitting decentration that has been discussed previously [20, 24–29]. However, the difference between PCCP and OCCP revealed the sole influence of lens fitting decentration on the reshaped corneal surface, which has received little attention.

A number of previous studies [17, 19–21, 24–27, 29, 35, 43] focusing on the corneal or ocular wavefront aberrations have uncovered a decrease in defocus ${C}_2^0$ and the increase in the vertical primary coma ${C}_3^{-1}$, horizontal primary coma ${C}_3^1$, primary spherical ${C}_4^0$, and RMS of high order wavefront aberrations owing to overnight OK lens wear. Similar results from the Zernike coefficients of corneal surface (see PCCP vs. PCCB in Table 1) were found in this study, which could account for the intended myopic correction [40, 44] and the induced wavefront aberrations.

Moreover, the sole influence of lens fitting decentration on corneal topography was revealed by comparing PCCP with OCCP, finding that lens fitting decentration could cause a further increase in RMS of 3rd order, RMS of 4th order and RMS of high order corneal surface Zernike coefficients but no significant change in ${C}_2^0$. It means that lens fitting decentration had an insufficient effect on the change of corneal spherical power, which may differ from the previous opinion of Yang et al. [18] who found that there was a definite tendency between refractive error and lens fitting decentration. Hiraoka et al. [19] also believed that fitting decentration was related to the refractive error. This controversy is the result of some critical factors, such as slightly diverse assessment parameters, distinct methods for the determination of fitting decentration, and different data processing methods of Zernike coefficients involved. The evidence supports our result about ${C}_2^0$ is that 98.1% subjects got the uncorrected visual acuity of logMAR 0.1 or better after 1-month treatment regardless of the diversity of lens fitting decentration.

Several studies have probed the relationship between lens fitting decentration and individual 3rd order Zernike coefficients of corneal aberrations [20] or ocular aberrations [19, 21, 35]. Here, based on the Zernike coefficients of corneal surface, the similar results obtained were that the horizontal primary coma ${C}_3^1$ was correlated with horizontal decentration (Fig. 4a and Fig. 7a) while the vertical primary coma ${C}_3^{-1}$ was correlated with vertical decentration (Fig. 5a and Fig. 8) both for (PCCP−PCCB) and (PCCP−OCCP). Furthermore, because of the higher level of correlation observed in Fig. 7a than that in Fig. 4a, the conventional analysis method based on (PCCP–PCCB) may underestimate the influence of horizontal lens fitting decentration on ${C}_3^1$. The same problem may arise with ${C}_3^{-1}$ by comparing Fig. 8 with Fig. 5a.

Remarkably, we found that perfect OK lens fitting might bring in an obvious change of ${C}_3^1$ statistically but not ${C}_3^{-1}$ as shown in OCCP vs. PCCB in Table 1. The main reason may be the corneal physiological difference along horizontal visual field [18, 45–47]. Apart from the relationship between principle meridian and 3rd order Zernike coefficients, ${C}_3^1$ was also found to be related to radial distance of decentration with a comparable level of correlation both for (PCCP – PCCB) and (PCCP – OCCP) (Fig. 3a and Fig. 6b), while ${C}_3^{-1}$ was related to radial distance of decentration only for (PCCP – OCCP) (see Fig. 6a). So, it may imply that the change of ${C}_3^1$ was caused by the shared effect of corneal reshaping and lens fitting decentration, while ${C}_3^{-1}$ was more easily affected by OK lens fitting decentration and could be compensated by corneal reshaping.

The same as the previous results [20, 26, 27], primary spherical ${C}_4^0$ underwent a statistical increase both by comparing PCCP with PCCB or comparing PCCP with OCCP (see Table 1). Figure 3b and Fig. 6c also demonstrate the positive correlation between ${C}_4^0$ and radial distance of decentration. However, the absence of statistical difference between OCCP and PCCB for ${C}_4^0$ (see Table 1) indicates that the change of ${C}_4^0$ might be caused by lens fitting decentration. Because of the complex interaction between ${C}_2^0$ and ${C}_4^0$ [40, 42, 44], the change in ${C}_4^0$ of corneal surface due to lens fitting decentration could bring in additional wavefront aberration of defocus.

It is interesting to find that horizontal secondary astigmatism ${C}_4^2$ in (PCCP – PCCB) was negatively correlated with horizontal decentration (Fig. 4b) and positively correlated with vertical decentration (Fig. 5b), while there was no significant change of ${C}_4^2$ either in OCCP vs. PCCB or in PCCP vs. OCCP (see Table 1). So, the change of ${C}_4^2$ may be caused by the composite effect of lens fitting decentration and corneal reshaping.

The correlation between horizontal secondary coma ${C}_5^1$ and horizontal decentration was found to be similar both for (PCCP – PCCB) and (PCCP – OCCP) as shown in Fig. 4c and Fig. 7b. We may deduce that the change of ${C}_5^1$ was solely induced by lens fitting decentration and insensitive to the interaction between corneal reshaping and lens fitting decentration. According to the statistical difference of vertical secondary coma ${C}_5^{-1}$ between OCCP and PCCB (see Table 1), the change of ${C}_5^{-1}$ was possibly associated with corneal reshaping but neutralized with lens fitting decentration. Additionally, the combination of ${C}_3^1$ and ${C}_5^1$ of cornea surface may cause horizontal coma-like wavefront aberration, since Applegate et al. [44] and Chen et al. [42] had pointed out that the individual Zernike coefficients with the same sign and angular frequency could be combined to interact.

When it came to the direction of lens fitting decentration, it was observed that temporal decentration, inferior decentration, and inferotemporal decentration accounted for 84.9%, 58.5% and 49.1% of all the fitted OK lenses, respectively. Such a tendency to inferotemporal decentration has been reported in several studies [18–20, 22, 35]. However, the different tendency to the superotemporal decentration might also be possible [21]. The temporal decentration was due to the steeper temporal side of cornea than the nasal side [18, 45–47]. The vertical decentration was presumed to be a composite result of eyelid tension, lens design and fitting technology [18–21].

One limitation of this study is that only corneal topography was quantified, the influence of fitting decentration on visual quality such as ocular wavefront or contrast sensitivity function was not taken into consideration. Another limitation is that fitting decentration was not phased in magnitude to clarify the levels of correlation with corneal Zernike coefficients which may provide the basis for fitting decentration classification in the clinic.

## Conclusions

The composite effect of corneal reshaping and lens fitting decentration of the OK lens on corneal topography was scrutinized using the conventional method based on comparing PCCP with PCCB. The sole influence of OK lens fitting decentration on the reshaped corneal surface was revealed by comparing PCCP with OCCP. OK lens fitting decentration within 1.5 mm can scarcely influence the change of corneal spherical power for myopia correction, but may significantly induce additional corneal high order Zernike coefficients including ${C}_3^{-1}$, ${C}_3^1$, ${C}_4^0$, and ${C}_5^1$.

## Abbreviations

LCSA: local corneal surface astigmatism
logMAR: logarithm of the minimum angle of resolution
OCCP: OK-lens-centered corneal Zernike coefficients of post-treatment
OK lens: orthokeratology lens
PCCB: pupil-centered corneal Zernike coefficients of baseline / pre-treatment
PCCP: pupil-centered corneal Zernike coefficients of post-treatment
TZ: treatment zone
